# Selective Sensing in Microbial Fuel Cell Biosensors: Insights from Toxicity-Adapted and Non-Adapted Biofilms for Pb(II) and Neomycin Sulfate Detection

**DOI:** 10.3390/mi14112027

**Published:** 2023-10-30

**Authors:** Abdelghani Ghanam, Sebastien Cecillon, Hasna Mohammadi, Aziz Amine, François Buret, Naoufel Haddour

**Affiliations:** 1 Univ Lyon, Ecole Centrale de Lyon, INSA Lyon, Université Claude Bernard Lyon 1, CNRS, Ampère, UMR5005, 69130 Ecully, France; abdelghani.ghanam@ec-lyon.fr (A.G.); francois.buret@ec-lyon.fr (F.B.); 2 Chemical Analysis and Biosensors Group, Laboratory of Process Engineering and Environment, Faculty of Science and Techniques, Hassan II University of Casablanca, B.P 146, Mohammedia 20000, Morocco; hasna.mohammadi@fstm.ac.ma (H.M.); a.amine@univh2m.ac.ma (A.A.)

**Keywords:** adapted biofilm, real-time detection, electrochemically active bacteria, microbial fuel cell, neomycin sulfate, toxicity biosensor, Pb(II) ions

## Abstract

This study introduces the utilization of self-powered microbial fuel cell (MFC)-based biosensors for the detection of biotoxicity in wastewater. Current MFC-based biosensors lack specificity in distinguishing between different pollutants. To address this limitation, a novel approach is introduced, capitalizing on the adaptive capabilities of anodic biofilms. By acclimating these biofilms to specific pollutants, an enhancement in the selectivity of MFC biosensors is achieved. Notably, electrochemically active bacteria (EAB) were cultivated on 3D porous carbon felt with and without a model toxicant (target analyte), resulting in the development of toxicant-resistant anodic biofilms. The model toxicants, Pb^2+^ ions and the antibiotic neomycin sulfate (NS), were deployed at a concentration of 1 mg L^−1^ during MFC operation. The influence of toxicity on biofilm growth and power production was investigated through polarization and power density curves. Concurrently, the electrochemical activity of both non-adapted and toxicity-adapted biofilms was investigated using cyclic voltammetry. Upon maturation and attainment of peak powers, the MFC reactors were evaluated individually as self-powered biosensors for pollutant detection in fresh wastewater, employing the external resistor (ER) mode. The selected ER, corresponding to the maximum power output, was positioned between the cathode and anode of each MFC, enabling output signal tracking through a data logging system. Subsequent exposure of mature biofilm-based MFC biosensors to various concentrations of the targeted toxicants revealed that non-adapted mature biofilms generated similar current–time profiles for both toxicity models, whereas toxicity-adapted biofilms produced distinctive current–time profiles. Accordingly, these results suggested that merely by adapting the anodic biofilm to the targeted toxicity, distinct and identifiable current–time profiles can be created. Furthermore, these toxicity-adapted and non-adapted biofilms can be employed to selectively detect the pollutant via the differential measurement of electrical signals. This differentiation offers a promising avenue for selective pollutant detection. To the best of our current knowledge, this approach, which harnesses the natural adaptability of biofilms for enhanced sensor selectivity, represents a pioneering effort in the realm of MFC-based biosensing.

## 1. Introduction

Environmental contamination stemming from anthropogenic activities, including mining, agriculture, and urbanization, consistently introduces a plethora of chemical contaminants (heavy metals, hydrocarbons, pesticides, drugs, endocrine disruptors, etc.) into water resources [1,2]. The consequent release of highly toxic waste streams poses significant threats to the functionality of downstream wastewater treatment plants (WWTPs), sometimes leading to irreversible damage [3,4]. Extended recovery periods for WWTPs have been observed post-toxicity shocks, and many WWTPs exhibit limited capability in effectively treating non-biodegradable compounds. As a result, they often act as concentrated pollutant discharge points, exacerbating ecological and human health risks [5]. For wastewater quality control, traditional analytical methods, such as liquid chromatography (HPLC), LC-tandem MS, immunoassays, atomic absorption spectrometry (AAS), and other spectrometric techniques, remain predominant [6]. While these techniques exhibit high sensitivity and accuracy in toxicity detection, their complexity, need for specialized equipment, and reliance on highly skilled personnel render them less suitable for on-site and real-time monitoring [7]. Moreover, these methods often fail to reflect the true biological impact of contaminants [8]. Stricter environmental regulations have necessitated the development of cost-effective and sensitive tools for on-site environmental analyses. Recent advancements emphasize the significance of on-line water quality monitoring, both in ensuring potable water safety and enhancing WWTP operations. In this realm, microbial fuel cell (MFC)-based biosensors have garnered substantial attention [9]. MFCs are bioelectrochemical systems that capitalize on microorganisms to transform chemical energy into electrical energy [10,11,12,13]. The biofilms, composed of electrochemically active bacteria (EAB), facilitate the oxidation of organic matter in the anodic compartment, producing protons and electrons. These electrons are then harnessed at the anode and relayed to the cathode via an external circuit, generating electricity [14,15]. The electrical output of MFCs is intrinsically linked to the metabolism of the EAB populating the anodic surface. Hence, the introduction of biotoxic compounds into wastewater can inhibit EAB metabolic processes, leading to a discernible decline in the MFC signal. Recent advancements in MFC-based biosensors have expanded their applications, ranging from monitoring water quality to detecting air pollutants [16,17]. For instance, Li et al. explored the use of a 2D smooth-anode-based MFC toxicity sensor for the enhanced detection of Pb^2+^ in wastewater [18]. Other studies by Haddour et al. and Cui et al. provided a comprehensive review of MFC-based biosensors, emphasizing their potential in biochemical oxygen demand (BOD) and toxicity detection [9,17]. Qiu et al. introduced a novel MFC biosensor capable of simultaneously detecting sodium acetate and glucose in mixed solutions [19]. Furthermore, Wang et al. developed a soil MFC-based self-powered cathodic biosensor for the sensitive detection of heavy metals [20]. Despite these advancements, challenges persist, particularly in enhancing the specificity of these biosensors. Most MFC biosensors detect overall toxicity, making it arduous to pinpoint the exact nature and origin of toxic substances in the water samples [9,17,21]. Addressing the specificity challenge, recent endeavors have either harnessed individual bacterial strains or genetically tailored strains for enhanced pollutant sensitivity [16,17]. Nevertheless, the financial implications of cultivating pure bacterial strains or their genetic modifications remain substantial. Few cost-effective alternatives are documented, save for some work emphasizing the role of external resistance in achieving relative specificity [22].

The present study proposes a novel configuration of a single-chamber air-cathode MFC-based biosensor designed for batch-mode operation. For targeted contaminant detection, EAB were cultivated on 3D carbon felt anodes in the presence of a toxic model (target to be analyzed), leading to the development of anodic biofilms resistant to the specific toxicant. Drawing inspiration from bioremediation techniques, this method promotes the enrichment of bacterial strains capable of metabolizing and degrading specific pollutants [19,20]. As a result, the presence of the target pollutant in a water sample might not inhibit the metabolic activity of the adapted biofilm, especially when contrasted with a non-adapted biofilm (biofilm formed in the absence of the pollutant). Thus, selective detection of pollutants can be achieved by simultaneously measuring signals from biosensors utilizing both adapted and non-adapted biofilms. This methodology yields more-stable signals from the adapted biofilm-based biosensor when exposed to pollutants, as opposed to biosensors based on non-adapted biofilms. To the best of our knowledge, such a differential measurement technique has never been previously described. In this study, Pb^2+^ ions and the neomycin sulfate (NS) antibiotic were chosen as representative toxicity models to assess their impact on anodic biofilm growth and power generation within MFCs. Upon maturation of both the non-adapted and adapted biofilms and the attainment of peak power outputs, the MFC reactors underwent evaluation as self-powered biosensors, exposed to varying concentrations of toxicants, utilizing the aforementioned differential measurement approach. The present study serves as an initial exploration to validate the feasibility of this novel methodology.

## 2. Materials and Methods

### 2.1. Chemicals and Materials

Neomycin sulfate (C_23_H_46_N_6_O_13_·xH_2_SO_4_) and lead (II) nitrate (Pb(NO_3_)_2_, ≥99.0%) were procured from Sigma Aldrich (St. Louis, MO, USA) and utilized without further purification. Additional materials included 10 wt% platinum on a carbon catalyst, 5 wt% Nafion, ≥99.5% 2-propanol, and sodium acetate (NaAc) as a carbon source. A polytetrafluoroethylene (PTFE) spray solution (3 in 1) was sourced from Castorama (Dardilly, France), and carbon felt (CF) was obtained from Graphitech (Froges, France). All other chemicals employed in this study were of analytical reagent grade. Primary wastewater effluent (7 mS cm^−2^), serving as an electrolyte, and anaerobic activated sludge, used as both an inoculum and a source of EAB, were collected from the Grand Lyon domestic wastewater treatment plant (Lyon, France). This sludge was fed with a NaAc (10 mM) medium. The primary wastewater effluent was utilized to prepare stock solutions of 80 mg L^−1^ neomycin sulfate (NS) and 80 mg L^−1^ Pb^2+^ ions. Initially, these stock solutions were introduced to the MFC reactors at varying pollutant concentrations (either NS or Pb^2+^) during startup to facilitate the growth and adaptation of the electroactive biofilm on pristine CF anodes. Subsequently, after the maturation of both non-adapted and pollutant-adapted biofilms, the MFC reactors were replenished with a new medium containing a 10 mM NaAc substrate, devoid of any toxicants. Thereafter, these reactors were exposed to incremental concentrations of pollutants using the prepared stock solutions.

### 2.2. Design and Construction of MFC-Based Biosensors

**Figure 1:**
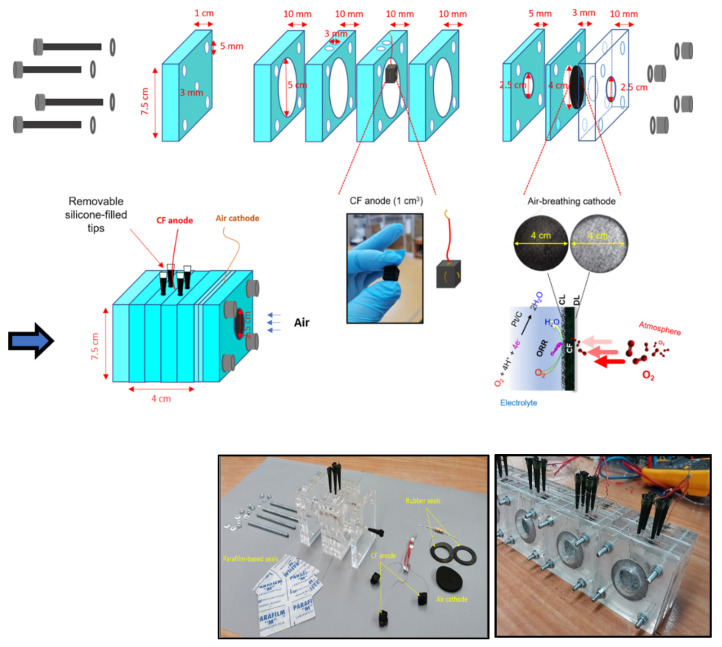
Schematic illustration of the components used for MFC biosensor construction and photos of pieces used in the final MFC biosensor assembly (**left**) and final forms of air-cathode, single-chamber MFC biosensors equipped with CF anodes and air cathodes after assembling (**right**).

Air-cathode, membrane-less, single-chamber MFCs, measuring 4 cm in length and 5 cm in diameter with an operational volume of approximately 80 mL, were fabricated from plexiglass, as depicted in Figure 1. The design and dimensions of the MFC components were drafted using Inkscape design software. Subsequent to design, the files generated using the Inkscape software were processed on a CO_2_ laser cutting machine to produce the 2D plexiglass components. Anodes, air cathodes, and homemade reference electrodes (Ag/AgCl, saturated KCl) were constructed by following the procedure described in the Supplementary Information. The anode, with dimensions of 1 × 1 × 1 cm^3^, was crafted from 3D porous pristine CF and positioned approximately 2 cm away from the air cathode within each MFC reactor. During the assembly of the MFC reactor, rubber and Parafilm seals were utilized to prevent potential water leakage at the interfaces between the air cathode and the plexiglass components, respectively. Additionally, holes were incorporated into all MFC reactors to facilitate filling, emptying, substrate addition, and pollutant introduction. These holes were subsequently sealed with removable silicone-plugged tips.

### 2.3. Inoculation and Start-Up of Pollutant-Adapted Biofilm-Based MFCs

**Figure 2:**
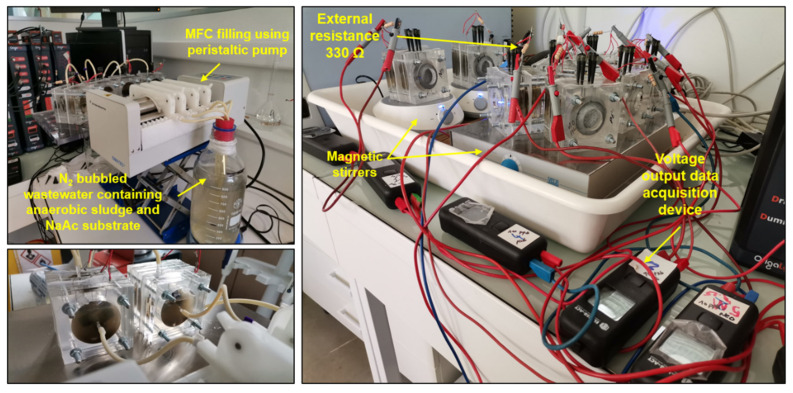
Pictures illustrating MFC filling with degassed wastewater containing anaerobic sludge and NaAc as carbon source using peristaltic pumping. MFC reactors, at the start point of experimentation, were subjected to different concentrations of pollutants (Pb^2+^ ions and NS) for biofilm growth/adaptation to pollutants.

Each MFC reactor was inoculated with an anaerobic digestion sludge concentration of 5 g L^−1^, combined with approximately 80 mL of fresh wastewater. This mixture was sourced from a municipal WWTP located in Lyon, France, and supplemented with 10 mM NaAc to serve as a carbon source. Prior to introduction into the MFC reactors, the wastewater effluent underwent a 15 m nitrogen bubbling process to eliminate dissolved oxygen. Subsequently, the MFC reactors were filled using a peristaltic pump and maintained in batch mode with continuous stirring at ambient temperature, as depicted in Figure 2. At the commencement of the operation, the MFC reactors were simultaneously exposed to varying concentrations of either NS or Pb^2+^ pollutants. Specifically, these pollutants were introduced separately into the MFC reactors at concentrations ranging from 0 to 1 mg L^−1^. The continuous exposure of the CF anodes to these toxicants during MFC operation facilitated the adaptation of the electroactive biofilms to their presence. The anode and cathode of each MFC reactor were interconnected via a stainless-steel wire, bridged by a 330 Ω external resistor (R_ext_). The employment of this specific R_ext_ was found to enhance the kinetics of anodic biofilm growth, fostering a rich and diverse microbial community [12]. This, in turn, optimized the capture of electrical energy during MFC operation.

### 2.4. Electrochemical Characterization

Upon achieving a consistent voltage output in all MFC reactors, the maturity of both non-adapted and pollutant-adapted electroactive biofilms on CF anodes was determined. Subsequently, the MFC operation mode transitioned to open circuit, and the reactors were interfaced with a potentiostat (OGS 500, Origalys, Rilleux-La-Pape, France) in a two-electrode configuration. The polarization and power density curves for all biofilm-based MFCs were then acquired using linear sweep voltammetry (LSV). During LSV measurements, the potentials were scanned from open-circuit potential (OCP) values to 0 V at a rate of 10 mV s^−1^. The power density (P, W m^−2^) curves were derived by multiplying the cell voltage with the current density (J, A m^−2^) using the equation P = V × I/S, where V and I denote the measured voltage and current, respectively. Both maximum power and current densities were normalized based on the geometric area (S) of the CF anodes. Furthermore, cyclic voltammetry (CV) was employed to analyze both non-adapted and pollutant-adapted biofilms (specifically to NS and Pb^2+^ ions) within a three-electrode MFC configuration. In this setup, CF anodes functioned as working electrode (WE), air cathodes as counter electrode (CE), and homemade Ag/AgCl electrodes as the reference electrode (RE). Analysis via CV provided insights into exoelectrogenic bacterial activity [22] and the potential impact of toxicity on electron transfer rate and/or biofilm growth and electrochemical activity. Notably, any alteration in the electrochemical behavior between biofilms cultivated in the absence or presence of toxicants could be discerned through CV. Such shifts might indicate whether the structural incorporation of toxicants within anode-colonizing biofilms impedes their metabolic activities related to the NaAc substrate. For all MFC reactors, the CV tests spanned a potential range from −800 mV to 700 mV (vs. Ag/AgCl) at a scan rate of 10 mV s^−1^.

### 2.5. Real-Time Toxicity Detection in Wastewater

Upon the maturation of biofilms, as evidenced by a consistent voltage output, power curves, and CV curves, all MFC reactors were replenished with a fresh wastewater medium containing solely 10 mM NaAc and 5 g L^−1^ activated sludge. Subsequently, the anode and cathode of each MFC biosensor were connected across an external resistor (R_ext_), which corresponded to the peak power generated by each respective MFC biosensor. The value of R_ext_ can be determined using the equation: P_max_ = J_max_ × V = R_ext_ × Jmax^2^. To introduce toxic shocks to the MFC biosensors, varying concentrations of toxicants were administered via a syringe through an aperture located at the top of the MFCs, as illustrated in (Figure S3). The subsequent alterations in the output voltage of each MFC biosensor in response to these toxic shocks were continuously monitored over time using a data logging instrument.

## 3. Results and Discussion

### 3.1. Toxicity Effect on Biofilm Growth and Power Generation

**Figure 3:**
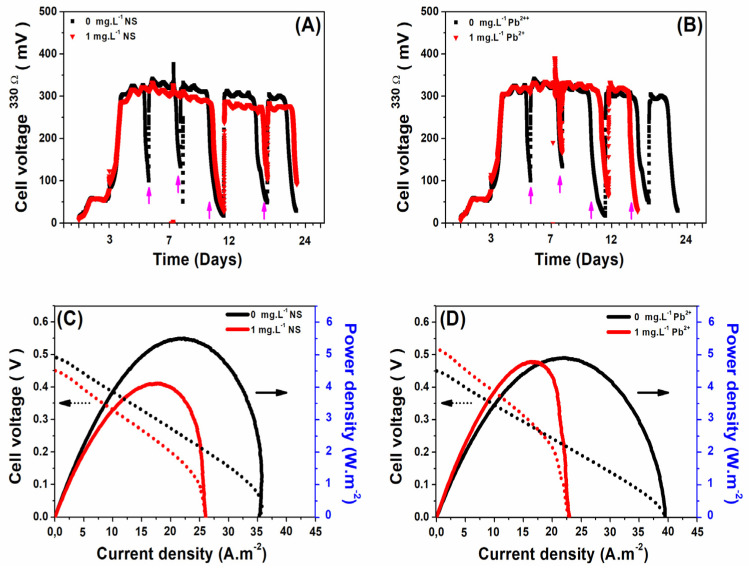
Voltage outputs produced during biofilm growth on CF anodes in MFCs operating separately without and with 1 mg L^−1^ of (**A**) NS antibiotic and (**B**) Pb^2+^ ions. The pink arrows represent the injection of 10 mM NaAc into the medium. Polarization and power density curves of non-adapted and adapted biofilm-based MFCs at different concentrations of (**C**) NS antibiotic or (**D**) Pb^2+^ ions were recorded using LSV. The biofilm on the CF anode in all MFC reactors reached maturity after 11 days of MFC operation. Scan rate, 10 mV s^−1^.

To assess the effects of specific pollutants on biofilm growth and electrochemical performance, the MFCs were subjected to two conditions: a control without pollutants and an experimental condition with a pollutant concentration of 1 mg L^−1^ (either NS antibiotic or Pb^2+^ ions). Figure 3A,B present the output voltages recorded over a 22-day operational span. These voltages provide a metric for evaluating the growth kinetics and maturation of anodic biofilms on CF anodes under both conditions. From the voltage–time profiles, it is evident that, after an initial seven-day period, all the MFCs achieved a stable output voltage, oscillating between 300 and 350 mV. This stabilization suggests the attainment of biofilm maturity, a finding consistent with previous observations from conventional air-cathode, single-chamber, bottle-type MFCs. The uniformity in voltage outputs across MFCs indicates that the introduction of pollutants, whether NS or Pb^2+^ ions, did not markedly alter the growth dynamics of the biofilms on the anodic surfaces. 

On the 11th operational day, after biofilm maturation confirmation via stable voltage outputs, LSV assessments were conducted at a rate of 10 mV s^−1^ using a two-electrode system configuration. The resulting polarization and power density curves for both non-adapted and pollutant-adapted biofilms are illustrated in Figure 3C,D. As delineated in Figure 3C, the incorporation of NS antibiotic during biofilm formation corresponded to around a 45% decrement in both the maximum power density and the maximum current density of the MFCs. This reduction can be attributed to the NS antibiotic’s potential inhibitory action on the metabolic processes of the electrogenic bacteria within the CF anode, subsequently slowing the consumption kinetics of the NaAc substrate. In contrast, as depicted in Figure 3D, while the maximum power density remained relatively invariant in the presence of Pb^2+^ ion concentrations, there was a discernible decrease (around 40%) in the maximum current density. These results suggest that although Pb^2+^ ions might hinder substrate transport to the anodic biofilm, the overall power generation remains largely unaffected.

### 3.2. Electroativity of a Mature Biofilm Adapted to Different Concentrations of Toxicity

To comprehensively assess the influence of toxicants on biofilm growth and electroactivity, CV tests were conducted in the MFCs. During these tests, a cyclic potential sweep was applied to the anodic biofilm relative to an external Ag/AgCl reference electrode (RE) at a scan rate of 10 mV s^−1^. Notably, these CV tests were conducted in the same effluents in which the biofilms were cultivated, containing 10 mM NaAc, in the absence and in the presence of 1 mg L^−1^ of toxicant. The initial CV recordings, taken after 3 days of MFC operation, exhibited no discernible redox peaks within the selected potential range (−0.8 V to +0.7 V), suggesting the absence of mature electroactive biofilms on the anode surfaces (Figures S4 and S5). However, by the seventh day, the voltammograms began to display characteristic faradaic peaks, indicative of the electrochemical behavior and electron transfer mechanisms inherent in the biofilms. By the 11th day, consistent voltammogram profiles emerged, corroborating the biofilm maturation hypothesis. 

**Figure 4:**
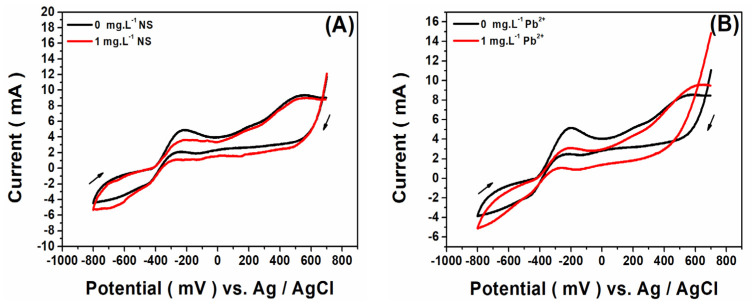
Cyclic voltammetry at 10 mV s^−1^ of MFC anodic biofilm reached maturity after 11 days of MFC operation without and with 1 mg L^−1^ of (**A**) NS antibiotic and (**B**) Pb^2+^ ions. Scan rate 10 mV s^−1^.

Figure 4 shows the CVs of MFC anodes recorded on the 11th days of the experiment both in the absence and presence of toxicants (NS or Pb^2+^ ions). The CVs of all the MFCs exhibited two peaks in both positive and negative potential regions. The sharp redox peaks observed at around −300 mV (vs. AgCl/Ag) and the oxidative peak at 400 mV (vs. AgCl/Ag) suggest that the direct electron transfer was the major EET mechanism, as previously described [23]. Based on previously reported values, the negative potential region corresponded to the mediated electron transfer of *Shewanella oneidensis* and/or heterogeneous electron transfer via the nanowires of *Geobacter sulfurreducens* EAB [24,25]. At positive potentials, the direct electron transfer was the major EET mechanism, and it could occur via the c-type cytochrome as in *Clostridium*, *Geobacter,* or *Shewanella* [26]. The similarities in the CVs of the anodes in all the MFCs suggest that the electron transfer mechanisms in the biofilms were not significantly influenced by the presence of the toxicants. However, the CVs displayed lower peak intensities with adapted biofilms, especially for peaks in negative region, in comparison with non-adapted biofilms. These results suggest that the presence of a toxicant during biofilm formation either reduces the catalytic electroactivity of the anodic biofilms or diminishes the amount of EAB in the biofilms, or both. A combined metaproteomics and metagenomics approach would offer a more comprehensive insight into both aspects.

### 3.3. Effect of Salinity on MFC Performance

Salinity is recognized as a pivotal factor influencing both the conductivity of the medium and the sensitivity of MFC-based biosensors [27]. To understand its implications, the influence of salinity on the output current of MFCs, with biofilms cultivated both in the absence and presence of toxicants (NS or Pb^2+^ ions), was examined. After the electroactive biofilms matured and the maximum electrical power generated by the MFCs stabilized, the existing solution was substituted with a fresh, oxygen-depleted medium. A multihead peristaltic pump facilitated the replacement process, ensuring that the integrity of the anodic biofilms remained undisturbed. This new medium solely comprised dehydrated activated sludge and the NaAc substrate. Following the stabilization of output voltages, a concentration of 60 mM NaCl (σ ≈ 7 mS cm^−2^) was introduced to all the MFCs. This concentration was deliberately chosen below the threshold observed in MFCs utilizing domestic wastewater as an inoculum to prevent any adverse effects on the biofilm’s physiology [28,29]. 

**Figure 5:**
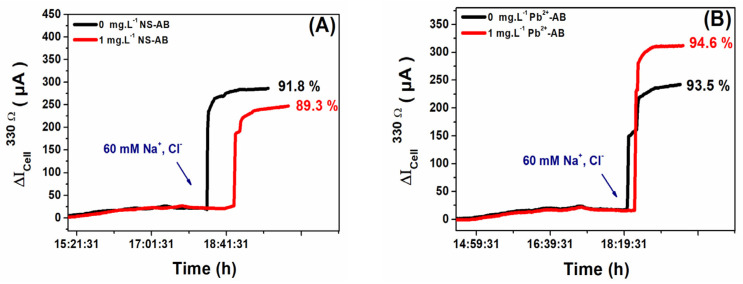
Effect of 60 mM (Na^+^, Cl^−^), σ ≈ 7 mS cm^−2^, on output currents of biofilm-based MFCs non-adapted and adapted to different toxicity concentrations: (**A**) NS antibiotic and (**B**) Pb^2+^ ions. These experiments were performed after the maturation/adaptation process of biofilms in a fresh medium with 10 mM NaAc substrate. The anode and cathode were connected across a 330 Ω external resistor.

Figure 5A,B illustrate the effect of NaCl addition on the output currents produced by biofilm-based MFCs non-adapted and adapted to 1 mg L^−1^ of NS antibiotic or Pb^2+^ ions. A notable increase of 35% and 33% in current output was observed in MFCs with biofilms exposed to NS and Pb^2+^ ions, respectively, after NaCl addition. All current–time trajectories exhibited consistent patterns after NaCl addition, suggesting that the MFC biosensor’s response to salinity remains uniform, irrespective of prior toxicant exposure. Otherwise, the process of biofilm adaptation to toxicity had no impact on the response of the MFC-based biosensor to the NaCl salt with respect to the non-adapted ones. 

Figure S6 further highlights the influence of salinity on the electrochemical activity of mature anodic biofilms. Employing the three-electrode setup, the CV measurements spanned a potential range of −1 V to +0.7 V vs. Ag/AgCl at a scan rate of 10 mV s^−1^. The results underscored a significant enhancement in the electrochemical activities of anodic biofilms following NaCl addition. For the subsequent application of these bioelectrochemical systems in biosensing or monitoring specific toxicants, it is imperative to maintain consistent salinity levels. Similarly, the response of the MFC biosensor should not be dependent on the NaAc substrate. Therefore, during toxicity sensing, the MFC biosensors should be at their maximum regardless of optimal NaAc substrate and NaCl salt concentrations. 

### 3.4. MFC Biosensor Response to Toxicity

**Figure 6:**
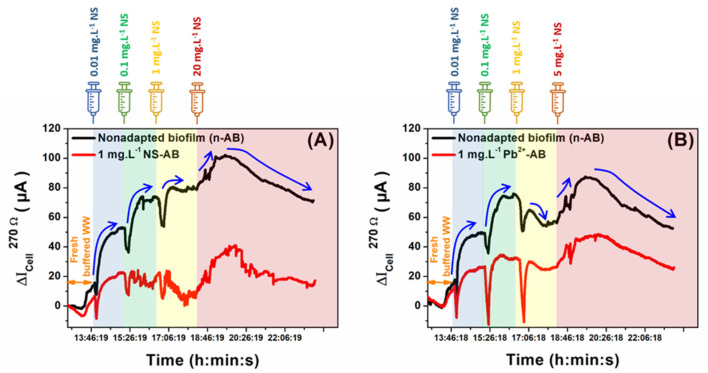
Change in output currents of mature electroactive biofilm-based MFCs non-adapted (black line) and adapted (red line) to 1 mg L^−1^ of NS or Pb^2+^ ions in fresh buffered wastewater with only 60 mM (Na^+^, Cl^−^), σ ≈ 7 mS cm^−2^, and 10 mM NaAc. Biofilms were challenged with successive additions of (**A**) NS in the concentration range 0.01–20 mg L^−1^ and (**B**) Pb^2+^ in the concentration range 0.01–5 mg L^−1^.

Following the effective operation of MFCs with electroactive biofilms cultivated in the absence and presence of various toxicant concentrations and having achieved a stable output voltage signal, the MFC reactors were evaluated as self-powered biosensors for detecting pollutants in wastewater. Control modes can influence MFC performance metrics, including sensitivity, dynamic range, recovery, and response times. Utilizing a fixed ER offers the advantage of monitoring the voltage drop across the resistor without the necessity for a potentiostat, external power supply, or reference electrode, thereby reducing operational costs [9]. This mode is particularly beneficial for the on-site monitoring of sudden toxicity events in wastewater, providing early alerts to WWTP personnel to take timely preventive measures. Initially, the MFC chambers were replenished with a fresh medium solution, comprising activated sludge, 10 mM NaAc substrate, and 60 mM NaCl. Once stable output voltage signals were established, the polarization and power density curves were derived using LSV at a scan rate of 10 mV s^−1^. This facilitated the determination of the maximum power densities for all the MFCs. Subsequently, the anode and cathode of each MFC biosensor were connected across a newly selected 270 Ω external resistor, chosen based on the maximum power density output of each MFC. Figure 6A,B depict the temporal variations in the output currents of the MFC biosensors, based on both non-adapted and toxicant-adapted (1 mg L^−1^ of NS or Pb^2+^ ions) mature electroactive biofilms. 

The biofilms were subsequently challenged with successive additions of NS in the concentration range 0.01–20 mg L^−1^ and Pb^2+^ in the concentration range 0.01–5 mg L^−1^ using a syringe. It is worth noting that each concentration was administered only once to avoid prolonged exposure of the non-adapted biofilm. This approach was taken to prevent the inadvertent development of resistance within the biofilm, which could compromise its sensitivity, especially when considering higher toxicant concentrations. As can be seen clearly for both NS and Pb^2+^ ion shocks, the responses of the non-adapted biofilms toward toxicity addition showed almost similar profiles. The introduction of either toxicant resulted in a comparable decline in the MFC’s current output, suggesting that non-adapted biofilms can detect a broad spectrum of pollutants, providing a holistic toxicity overview of both anticipated and unanticipated contaminants in real samples. This observation aligns with prior studies indicating that non-adapted biofilms cannot discern specific toxic shocks [9]. 

Moreover, during toxicant exposures, non-adapted biofilm-based MFC biosensors displayed a rapid current output reduction post-toxicant introduction, followed by a return to an elevated baseline. This baseline shift was more pronounced at lower concentrations and diminished at higher levels. Such results might be attributed to the fact that the minimal toxicant concentrations were insufficient to inhibit the entire catalytic activity of the bacterial population in the anodic biofilms. Furthermore, the elevated baseline during low toxicant exposure might be indicative of the acclimatation process of non-adapted biofilms to the toxicant, leading to enhanced bacterial activity for energy production. This acclimatation process has been previously described when exposing anodic biofilms to repetitive acute toxicity event tests, increasing the pollutant degradation activity of biofilms and reducing their sensitivity of toxicity detection [30]. 

On the contrary, anodic biofilm-based MFC biosensors adapted to 1 mg L^−1^ NS or Pb^2+^ ions showed different MFC output current profiles during toxicity shocks. Indeed, the current production obtained with the MFC biosensor based on the NS-adapted biofilm was lower than the non-adapted one during shocks with NS. This result could prove that the anodic biofilm acclimated and formed with 1 mg L^−1^ NS has developed bacterial resistance to NS shocks. Unlike NS shocks, drops in the current output during Pb^2+^ shocks of the MFC biosensor based on the Pb^2+^-adapted electroactive biofilm, were higher than those provided by the non-adapted MFC. These results may prove that the anodic biofilm’s adaptation to Pb^2+^ ions improved the sensitivity of the MFC biosensor to Pb^2+^ biosensing in wastewater. 

In summary, while non-adapted biofilm-based MFC biosensors exhibited similar current–time profiles for both NS and Pb^2+^ exposures, distinct profiles were observed for the MFC biosensors with Pb^2+^ and NS-adapted biofilms. However, by adapting the anodic biofilm to a targeted toxicant, distinct and characteristic current–time profiles can be generated during toxicant exposures. Based on these profiles, biosensor responses to such a toxicant can be easily distinguished, thereby achieving specific and selective detection of toxicants using MFC-based biosensors. The concept of anodic biofilm acclimation/adaptation to toxicants can be leveraged in a differential sensing approach, facilitating the development of biosensors to monitor the overall toxicity of environmental contaminants and distinguish various analytes in complex solutions.

## 4. Conclusions

This comprehensive evaluation of MFC reactors as self-powered biosensors for detecting pollutants in wastewater yielded significant insights. This study demonstrated that the electroactivity of biofilms, both non-adapted and adapted to specific toxicants, plays a pivotal role in the MFC’s sensitivity and response to pollutants. The influence of salinity on MFC performance further underscored the importance of environmental factors in determining the efficacy of these biosensors. The ability to track voltage drops using fixed external resistors, without the need for additional equipment, not only reduces operational costs but also enhances the feasibility of on-site monitoring, providing timely alerts for wastewater treatment plant operations. Furthermore, this study highlighted the potential of biofilm adaptation as a strategy to enhance the specificity and selectivity of MFC biosensors. While non-adapted biofilms exhibited a generalized response to a wide range of pollutants, adapted biofilms showcased a more targeted and pronounced response to specific toxicants. This distinction in response profiles offers a promising avenue for the development of MFC biosensors that can not only monitor overall toxicity but also differentiate between various contaminants in complex solutions. 

In light of the findings of this study, a deeper understanding of the microbiological structure of biofilms during their growth in the presence of pollutants emerges as a critical avenue for future research. By delving into the intricate interactions and adaptive mechanisms at the microbial level, it is possible to gain insights into the specific bacterial species that thrive under toxicant exposure and those that are inhibited. Advanced techniques, such as fluorescent analysis and metagenomic sequencing, can reveal the distribution of live vs. dead bacteria, as well as the compositional shifts in microbial communities. Such studies can not only validate the electrochemical findings of biofilm adaptation but also shed light on the broader ecological implications of pollutant exposure. Investigating the microbiological intricacies will undoubtedly enrich the understanding of biofilm dynamics, offering a more holistic view of their resilience and adaptability in the face of environmental challenges. In conclusion, the findings of this study highlight the potential of MFC-based biosensors as robust tools for the real-time monitoring of wastewater pollutants. The insights gained pave the way for further research aimed at optimizing these systems for broader applications in environmental monitoring and pollution control.

## Data Availability

The data presented in this study are available in Supplementary Materials.

## Supplementary Materials

The following supporting information can be downloaded at https://www.mdpi.com/article/10.3390/mi14112027/s1, Figure S1: Silver wires used to prepare Ag/AgCl electrodes by immersing them in a solution of 50 mM FeCl_3_⋅6H_2_O to form AgCl films on the silver wires; Figure S2: (A) Glass Pasteur pipette plugging with ion-conducting agarose hydrogel. (B) Cooling of the hydrogel-plugged glass Pasteur pipettes at one end by immersing them in a cold KCl solution. (C) Pasteur pipette closure with silicone and storage in saturated KCl solution; Figure S3: Injection of the targeted pollutant (NS or Pb^2+^ ions) in the MFC biosensor containing fresh wastewater solution with 10 mM NaAc; Figure S4: CV curves for different biofilm growth periods on CF anodes describing the electrochemical behavior of a non-adapted electroactive biofilm and a biofilm adapted to (A) 0 and (B) 1 mg L^−1^ Pb^2+^ ions in MFC reactors. Scan rate 10 mV s^−1^; Figure S5: CV curves for different biofilm growth periods on CF anodes describing the electrochemical behavior of a non-adapted electroactive biofilm and a biofilm adapted to (A) 0 and (B) 1 mg L^−1^ NS antibiotic in MFC reactors. Scan rate 10 mV s^−1^; Figure S6: CV curves recorded in fresh wastewater without (a) and with (b) 60 mM Na^+^, Cl^−^, σ ≈ 7 mS cm^−2^, on different mature biofilms already formed on CF anodes and non-adapted and adapted to 1 mg L^−1^ (A) NS antibiotic and (B) Pb^2+^ ions in MFC reactors. Scan rate 10 mV s^−1^. References [31,32] are cited in Supplementary Materials.
