# Supplementary material for: Selective Sensing in Microbial Fuel Cell Biosensors: Insights from Toxicity-Adapted and Non-Adapted Biofilms for Pb(II) and Neomycin Sulfate Detection
Source: Micromachines (Basel). 2023 Oct 30;14(11):2027. doi: 10.3390/mi14112027 (PMC10673119; doi:10.3390/mi14112027)
Supplement: Supplementary file 1 [file micromachines-14-02027-s001.zip › micromachines-2661270-supplementary.pdf]

Supplementary Materials

Selective Sensing in Microbial Fuel Cell Biosensors: Insights from Toxicity-Adapted and Non-Adapted Biofilms for Pb(II) and Neomycin Sulfate Detection

Abdelghani Ghanam [1,2], Sebastien Cecillon [1], Hasna Mohammadi [2], Aziz Amine [2], François Buret [1] and Naoufel Haddour [1,*]

1 Univ Lyon, Ecole Centrale de Lyon, INSA Lyon, Université Claude Bernard Lyon 1, CNRS, Ampère, UMR5005, 69130 Ecully, France; abdelghani.ghanam@ec-lyon.fr (A.G.); sebastien.cecillon@ec-lyon.fr (S.C.); francois.buret@ec-lyon.fr (F.B.)

2 Chemical Analysis and Biosensors Group, Laboratory of Process Engineering and Environment, Faculty of Science and Techniques, Hassan II University of Casablanca, B.P 146, Mohammedia 20000, Morocco; hasna.mohammadi@fstm.ac.ma (H.M.); a.amine@univh2m.ac.ma (A.A.)

* Correspondence: naoufel.haddour@ec-lyon.fr; Tel.: +33-4-72-18-61-12

Anode and air-cathode fabrication

The pristine carbon fiber (CF) was subjected to a sequential pretreatment involving three distinct solvents (ethanol, acetone, and water), with each solvent undergoing 15 min of ultrasonication. Subsequently, the CF was dried at 60 °C for 3 h and then cut into 1 x 1 x 1 cm^3 cubes. In the final step, all fabricated anodes were connected using stainless steel wire to establish electrical contact with the external circuit. With these preparations completed, the anodes are now ready for placement into the MFC systems. The cathode was prepared with a PTFE coating and a 5% platinum catalyst, following the procedure outlined by Cheng et al. [1].

Home-made reference electrode fabrication

Due to the limitations of commercial Ag/AgCl reference electrodes (RE) in terms of size, a cost-effective, miniaturized Ag/AgCl (saturated KCl) RE was fabricated. The fabrication process was executed in three distinct phases: (i) Ag/AgCl Formation: Silver wires were partially immersed in a 50 mM $FeCl_3.6H_2O$ solution for a duration of 1 min (Figure S1). This immersion facilitated the formation of an AgCl film on the silver wires, in accordance with the specified redox reactions [2]. Subsequently, the silver wires, now coated with the AgCl film, were transferred to a saturated KCl solution. (ii) Preparation of ion-conducting agarose hydrogel: This phase involved the creation of a conductive hydrogel to act as a salt bridge at one end of a Pasteur pipette. The hydrogel was formulated by dispersing 1% (w/v) agarose in a saturated KCl solution (0.2 g in 20 mL). This mixture was subjected to microwave irradiation for a total of 1 min (in two 30-second intervals) to ensure complete agarose dispersion. The resultant dispersion was then placed on a preheated hotplate set at 80 °C and stirred vigorously. Concurrently, aliquots of the solubilized agarose solution were injected into one end of Pasteur pipettes and subsequently cooled in a saturated KCl solution. (iii) Assembly of the reference electrode: Once the hydrogel solidified, the pipettes were filled with saturated KCl. The previously prepared Ag/AgCl electrodes were then inserted, yielding Ag/AgCl (saturated KCl) REs analogous to commercial variants (Figure S2). The opposite ends of the Pasteur pipettes were sealed using silicone and left to dry at room temperature prior to their deployment in MFC systems. The potential of this home-made reference electrodes was measured against commercial AgCl/Ag reference electrodes, and only those electrodes exhibiting a shift of less than 10 mV were retained. This potential difference was noted for each electrode and taken into account to adjust the potential values presented.

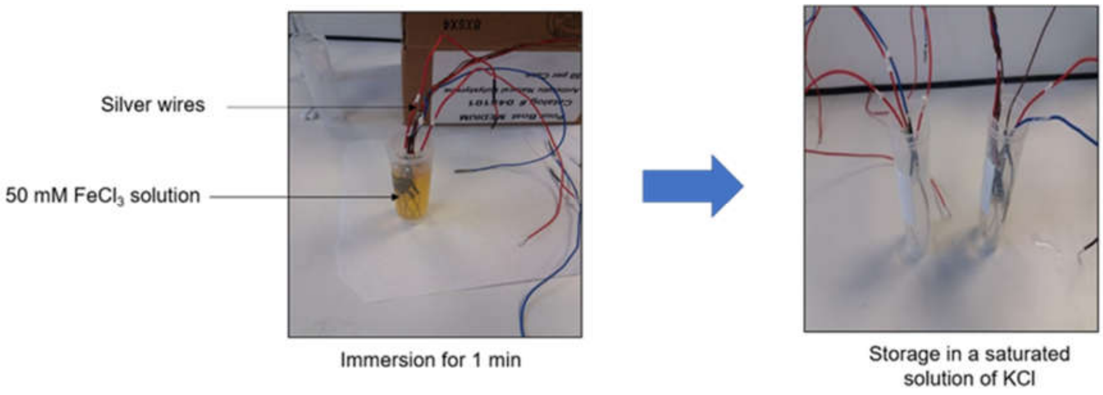

Figure S1. Silver wires used to prepare Ag/AgCl electrodes by immersing them in a solution of 50 mM $FeCl_3 \cdot 6H_2O$ to form AgCl films on the silver wires.

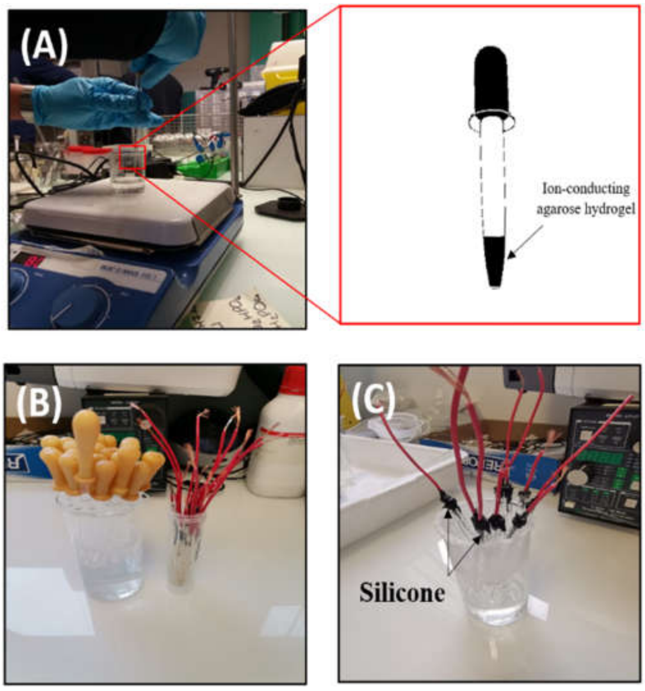

Figure S2. (**A**) Glass Pasteur pipettes plugging with ion-conducting agarose hydrogel. (**B**) Cool the hydrogel-plugged glass Pasteur pipettes at one end by immersing them in a cold KCl solution. (**C**) Pasteur pipette closure with silicone and stored in saturated KCl solution.

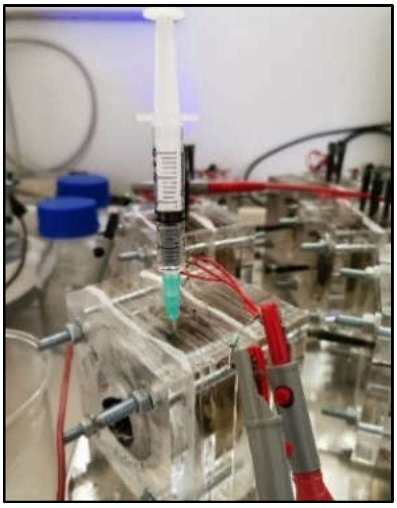

Figure S3. Injection of the targeted pollutant (NS or Pb^{2+} ions) in the MFC biosensor containing fresh wastewater solution with 10 mM NaAc.

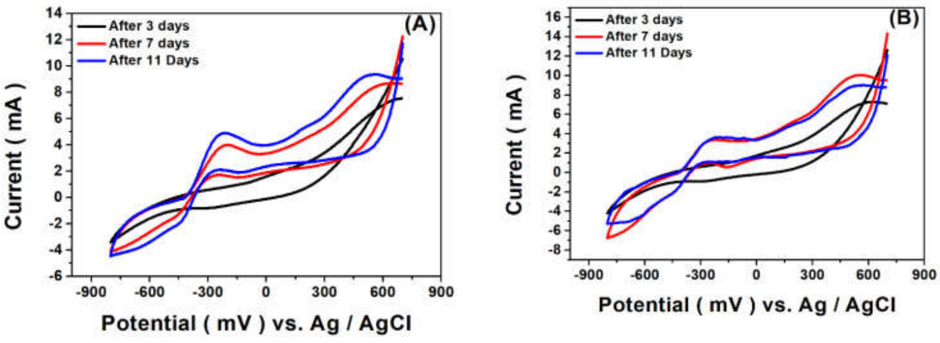

Figure S4. CV curves for different biofilm growth periods on CF anodes describing the electrochemical behavior of nonadapted electroactive biofilm and adapted to (**A**) 0 and (**B**) 1 mg L^{-1} Pb^{2+} ions in MFC reactors. Scan rate 10 mV s^{-1}.

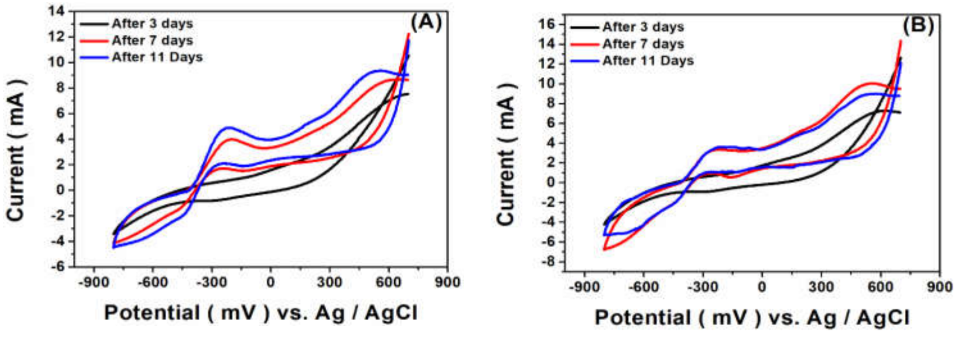

Figure S5. CV curves for different biofilm growth periods on CF anodes describing the electrochemical behavior of nonadapted electroactive biofilm and adapted to (**A**) 0 and (**B**) 1 mg L^{1} NS antibiotic in MFC reactors. Scan rate 10 mV s^{-1}.

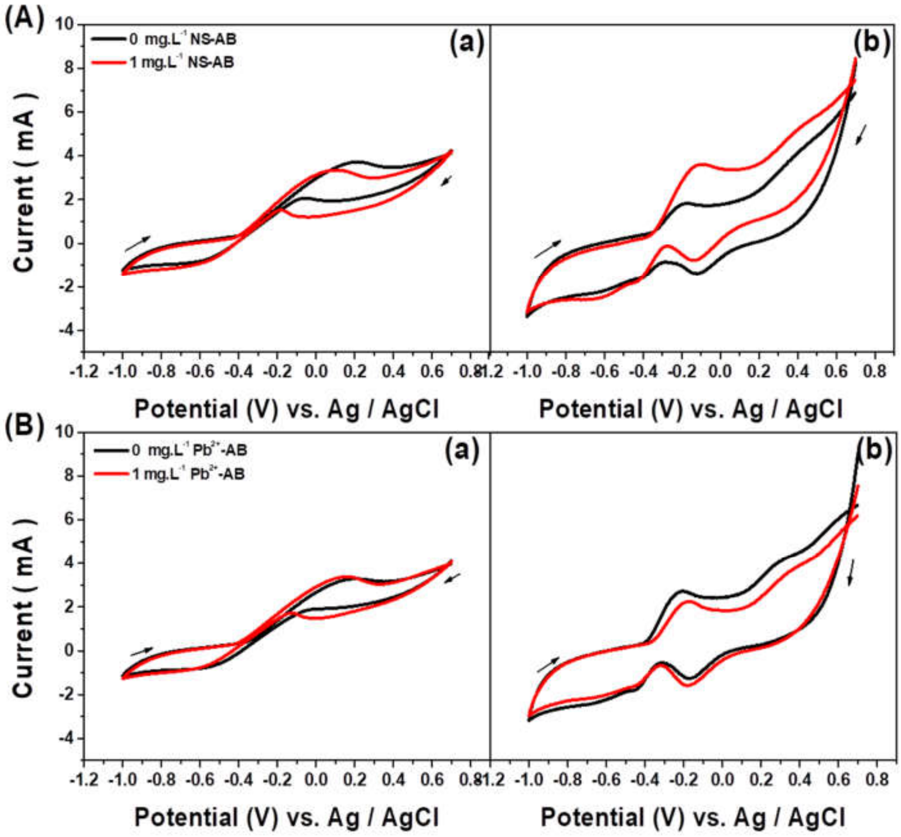

Figure S6. CV curves recorded in fresh wastewater without (a) and with (b) 60 mM Na+, Cl-, σ ≈ 7 mS cm^2, on different mature biofilms already formed on CF anodes and nonadapted and adapted to 1 mg L^1 (**A**) NS antibiotic and (**B**) Pb^{2+} ions in MFC reactors. Scan rate 10 mV s^1.

References

1 Cheng, S.; Liu, H.; Logan, B.E. Increased Performance of Single-Chamber Microbial Fuel Cells Using an Improved Cathode Structure. *Electrochem. commun.* **2006**, *8*, 489–494, doi:10.1016/j.elecom.2006.01.010.

2 Feng, C.; Li, F.; Liu, H.; Lang, X.; Fan, S. A Dual-Chamber Microbial Fuel Cell with Conductive Film-Modified Anode and Cathode and Its Application for the Neutral Electro-Fenton Process. *Electrochim. Acta* **2010**, *55*, 2048–2054, doi:10.1016/j.electacta.2009.11.033.
